# Individual patient disease activity expression profiles modify the relation between disease activity and structural damage progression in RA

**DOI:** 10.1016/j.ero.2026.100243

**Published:** 2026-09-30

**Authors:** Chiara Ripepi, Bram van Es, Janneke Tekstra, Julia Spierings, Jacob M. van Laar, Saskia Haitjema, Paco M.J. Welsing

**Affiliations:** Rheumatology and Clinical Immunology, University Medical Center Utrecht, Utrecht, The Netherlands

## Abstract

**Objectives:**

Disease activity in rheumatoid arthritis (RA) is commonly assessed using composite indices such as the Disease Activity Score in 28 Joints (DAS28). However, individual disease activity markers can be more or less pronounced in patient subgroups, and markers beyond disease activity indices may contribute to relevant disease activity patterns. This study aimed to identify patient subgroups with ‘disease activity expression profiles’ and evaluate their relevance.

**Methods:**

Routine-care data at University Medical Center Utrecht were extracted. Seventeen parameters associated with disease activity were included in a clustering analysis, with the optimal number of clusters determined using fit statistics and clinical interpretation. Using random effects modelling, the association between DAS28 and radiographic progression was assessed, including whether this relation was modified by expression profile.

**Results:**

Among 1167 patients with RA, three disease activity expression profiles were identified: (i) Systemic inflammatory (n = 330), characterised by high swollen joint counts and elevated inflammatory markers; (ii) Symptom dominant (n = 191), marked by high patient-reported activity and tender-swollen joint discrepancy; and (iii) Low-typical (n = 646), with low-to-moderate disease activity and less divergent marker expression. Higher DAS28 was associated with radiographic progression (odds ratio = 1.40 [1.03-1.92], *P* = .032). This relation was confined to the Low-typical and Systemic inflammatory subgroups, whereas absent in the Symptom-dominant group (*P* interaction = .015). Patients in the Systemic inflammatory subgroup had a higher progression risk.

**Conclusions:**

Derived disease activity expression profiles influence the predictive value of DAS28. Taking identified expression profiles into account may improve disease activity interpretation and support tailored monitoring and management in RA.

**WHAT IS ALREADY KNOWN ON THIS TOPIC**

• The DAS28 is widely used to guide rheumatoid arthritis management, but similar scores can reflect different combinations of systemic inflammation, joint findings, and patient-reported symptoms.

**WHAT THIS STUDY ADDS**

• Three distinct and largely stable disease activity expression profiles were identified: Systemic inflammatory, Symptom-dominant, and Low-typical. These profiles modified the relationship between DAS28 and radiographic progression: this relationship was absent in the Symptom-dominant profile, while the Systemic inflammatory profile showed a higher progression risk independent of DAS28.

**HOW THIS STUDY MIGHT AFFECT RESEARCH, PRACTICE OR POLICY**

• Considering disease activity expression profiles alongside DAS28 may improve interpretation of disease activity, risk stratification, and tailored monitoring for RA.

## INTRODUCTION

Rheumatoid arthritis (RA) is a chronic systemic autoimmune disease primarily characterised by persistent joint inflammation, often leading to joint damage and long-term disability. Extra-articular involvement is common as RA can affect multiple organ systems beyond the joints, contributing to a broad spectrum of clinical manifestations [1]. Disease activity in RA is multidimensional and lacks a universally accepted gold standard; consequently, researchers and clinicians often rely on disease activity composite indices that integrate clinical and laboratory parameters. The standardisation of disease activity measurements has been instrumental in clinical research and has facilitated the development of the treat-to-target (T2T) strategy, a paradigm in which therapeutic decisions are guided by targeting predefined disease activity levels, typically low disease activity (LDA) or remission [2]. Several validated indices are routinely used, such as the Disease Activity Score in 28 Joints (DAS28), the Clinical Disease Activity Index (CDAI), and the Simplified Disease Activity Index [[3], [4], [5]]. T2T has significantly improved long-term outcomes in RA, reducing disease progression and increasing the likelihood of achieving remission or LDA [2,6,7]. However, despite its widespread adoption, several questions remain unresolved. Some components of indices can be subjective, hard to assess, or be influenced by other patient characteristics, resulting in either overestimated or underestimated disease activity in certain cases [8,9]. Also, it is unclear which specific target should be prioritised (eg, remission or LDA) and which disease activity index should be used for optimal treatment guidance. In addition, using standardised targets assume a uniform relationship between disease activity indices and patient outcomes, potentially neglecting crucial interindividual variability in disease expression. This assumption may not be justified in RA, for which several sub- or phenotypes have been proposed [10,11]. The same disease activity score might reflect distinct pathophysiological mechanisms or symptomatic burdens in different patients, which may indicate a need for a more personalised approach to disease monitoring, which could help in determining the most optimal disease management for each patient.

Composite disease activity indices typically include laboratory measures such as erythrocyte sedimentation rate (ESR) and C-reactive protein (CRP), clinical assessments of tender and swollen joint counts (TJC28 and SJC28), and physician and/or patient-reported global assessments, (often on a visual analogue scale for Global Health [VAS-GH]) [12]. Beyond these, specific subindices (eg, the TJC28-SCJ28 difference and DAS28-P, the proportion of DAS28 attributable to patient-reported components [TJC and VAS-GH]) have been proposed to refine interpretation of objective disease activity [13,14]. Furthermore, blood-based inflammatory ratios such as the neutrophil-to-lymphocyte ratio (NLR), platelet-to-lymphocyte ratio (PLR), and lymphocyte-to-monocyte ratio (LMR) have emerged as objective biomarkers associated with inflammatory burden, disease severity, and treatment response [[15], [16], [17]]. These markers are attractive not only because they are routinely collected, machine-derived, and potentially less susceptible to observer variation, but also because they may provide additional information on inflammatory status beyond the typically used disease activity index components.

Therefore, this study aims to explore whether individual disease activity expression profiles exist in RA and whether these profiles may provide clinically meaningful insights into patient subgroups with different characteristics, disease progression, treatment, or monitoring need. To this aim, we will also assess the potential impact of envisioned profiles on the known relationship between disease activity, as measured by the DAS28 index, and structural joint damage progression during follow-up. Ultimately, this research may contribute to personalised treatment strategies and improved patient outcomes.

## METHODS

Structured and unstructured pseudonymised routine-care data from patients with RA treated at the Department of Rheumatology & Clinical Immunology of the University Medical Center (UMC)Utrecht were extracted from electronic health records via the Utrecht Patient Oriented Database (UPOD). UPOD data acquisition and management are in accordance with current regulations concerning privacy and ethics as previously described [18]. The institutional review board of the UMC Utrecht waived the need for informed consent as only pseudonymised data of a large sample size were used for this study. Experienced UPOD data managers followed the Quality Management System (ISO 9001)-certified multidisciplinary workflow to obtain the data for the study. In brief, this included removing individuals who opted out of reuse of their data in research, inclusion of approved laboratory values according to the laboratory (ISO 15189), as well as double pseudonymisation before preprocessing by the researcher.

The study population comprised patients with RA according to the 10th revision of the International Classification of Diseases codes. Patients with the classification M05.X and M06.X (seropositive RA and other RA, respectively) and who had at least 2 X-rays of hands and feet taken were followed from 2003 up to 2022. Subsequently, the following data were extracted:

• Age (at time of RA diagnosis) and biological sex
• Clinical chemistry lab parameters (CRP, ESR, alanine aminotransferase [ALAT]) and haematology lab parameters (haemoglobin [Hb], systemic immune-inflammation index (SII), NLR, PLR, LMR).

The haematological lab parameters are routinely available in UPOD for all patients for whom 1 or more components of the complete blood count (CBC) have been requested (eg, Hb), as they are all routinely measured by the Abbott Celldyn Sapphire machines (Abbott Hematology) and subsequently systematically collected in UMC Utrecht.

• Routinely collected RA-related measurements were obtained. These included 28 joint counts for swelling and tenderness (SJC28/TJC28), and derived measures such as tender-swollen difference (TSD) and DAS28-P, swollen and tender joint counts for large joints, shoulder, elbow and knee only (LJI_SJC/LJI_TJC) and general health scored by patients on a VAS (VAS-GH) [19].
• Radiology reports (unstructured, text data) were also extracted from the same period. Reports were processed with a text-mining pipeline combining pattern-matching, weak supervision (Snorkel), active learning (Prodigy), and named-entity classification (MedCAT v1) to classify the presence/absence of progression. We finally fine-tuned MedRoBERTa.nl (a transformer model pretrained for the clinical domain) to identify progression/no-progression. In a subset of 70 reports labelled by 2 experts (JT and PMJW) consensus served as the reference, obtained with Kappa-Cohen=0.5, comparable to interannotator agreement [20].
• RA letters were mined for comorbidities using MedCAT v1; given a seeding list of terms/ICD10 codes describing the comorbidities, relevant medical concepts were identified in the UMLS ontology on the basis of similarity between RotatE embeddings [21].

### Identification of disease activity expression profiles

The hypothesis was that specific disease activity expression profiles-subgroups with relatively high or low expression of specific disease activity components exist. To classify patients with RA into such disease activity expression profiles, all disease activity component scores, derived indices, and laboratory markers were analysed. K-means clustering (using Euclidean distance) as the most often used technique for subgrouping or phenotyping patients was applied to the above-mentioned markers [22,23]. The number of clusters (k) was determined by peak silhouette scores and an increase in gap statistics across solutions (k = 2-5), combined with clinical interpretability of candidate subgroups [24,25]. To account for multiple visits within patients, in the cluster analysis, all variables were first averaged per patient over time to obtain independent observations for analysis.

Sensitivity analysis repeated clustering at the observation level (ie treating each visit as an independent observation). In this analysis, we also assessed whether derived clusters (expression profiles) should be seen as patients’ traits rather than a time-varying patient state by calculating the proportion of observations classified according to the most occurring cluster within each patient. We predefined that the most occurring cluster in patients should at least be present in 75% of observations be present in at least 75% of patients (ie Q3 of proportion cluster membership ≥0.75). In a second sensitivity analysis, to emphasise relative expression, we derived a ranking score by dividing the absolute z-score of each component by the z-score of the DAS28 (ESR-based, 3-variable definition) and subsequently performed the cluster analysis on these scores.

### Interpretation of disease activity expression profiles

The relative value of individual parameters within clusters relative to their overall mean and general patient and disease characteristics of derived cluster solutions were described. Furthermore, to evaluate the contribution of individual parameters to cluster separation, a random forest classifier was trained with cluster membership as the outcome, and mean decrease in accuracy and Gini importance were computed for each parameter [26]. This analysis was used to rank variable importance in cluster separation and was distinct from the multinomial regression model used to evaluate first-visit predictors of cluster membership.

### Association of disease activity expression profiles with radiographic progression of joint damage

The selected disease activity expression clusters were further scrutinised for clinical relevance by evaluating their relationship with progression of joint damage and specifically with the (known) longitudinal relation of DAS28 with progression of joint damage [27,28]. Using mixed-model logistic regression analysis (with a random intercept at the patient level), the association of repeated average DAS28 values between X-ray assessments and the repeated presence/absence of progression of joint damage was assessed. Subsequently, the patients’ disease activity expression profile membership was added to the model to assess any added effect on joint damage progression risk. Finally, the interaction between disease activity expression profile membership and DAS28 values was added to investigate whether cluster membership modified the relation between DAS28 and progression of joint damage. All analyses were corrected for follow-up time, duration between 2 assessments of radiographic progression (to correct for varying duration between X-rays), as well as age at diagnosis, sex, and baseline presence of radiographic progression as time-independent covariates.

### Prediction of cluster membership

We evaluated whether cluster membership can be predicted at first visit from patient and disease characteristics using multinomial logistic regression. Predictors included baseline DAS28, its components, and derived indices, and the laboratory markers and ratios NLR, ESR, CRP, Hb, PLR, LMR, and SII. Model performance was summarised as the overall percentage of correctly classified patients and compared with the no-information rate, defined as the accuracy obtained by assigning all patients to the largest cluster. We assessed performance using 5-fold cross-validation with stratification by cluster [29].

## RESULTS

Data from 1167 patients with RA were extracted. The cohort was predominantly female (73.2%), with a mean age of 55.5 years at the first visit (SD = 14.7). Patients were followed for an average of 15 years (SD = 6.3) with a varying number of follow-up visits at which at least 1 disease activity parameter was recorded (totalling 134,701 visits). In addition, 7200 radiographic images were scored for joint damage and progression, with 59.5% of patients exhibiting existing joint damage on their first recorded X-ray in UMC Utrecht (Table 1). The mean disease activity (DAS28) over the observation period was 3.07 (SD = 0.97).

**Table 1:** Demographic and clinical characteristics of RA, categorised by their cluster affiliation

	Total cohort (1167)	Low-typical DA (646)	Systemic inflammatory DA (330)	Symptom-dominant DA (191)
Age	55.59 ± 14.69	53.02 ± 14.13	61.76 ± 14.70	53.67 ± 13.37
Female sex, n (%)	846 (72.5)	471 (72.9)	225 (68.2)	150 (78.5)
Baseline damage (%)	683 (60.7)	365 (57.8)	231 (74.3)	87 (47.8)
Rheumatoid Factor positive n (%)	1008 (88.3)	440 (92.1)	201 (88.2)	137 (88.4)
Follow-up years	14.97 ± 6.27	15.39 ± 5.98	14.87 ± 6.63	13.72 ± 6.43
Follow-up visits, median (IQR)	84 [53, 148]	76.50 [51, 113.75]	105 [66, 194.75]	110 [47, 216]
Years from Diagnosis to start of follow-up, median (IQR)	1.2 [0.3, 3.7]	1.4 [0.5, 4.9]	0.6 [0.1, 2.4]	1.1 [0.3, 3.3]
Number of X-rays, median (IQR)	4.00 [2.00, 8.00]	4.00 [2.00, 7.75]	4.00 [1.00, 8.00]	4.00 [1.00, 8.00]
DAS28-ESR	3.07 ± 0.97	2.62 ± 0.73	3.41 ± 0.84	4.04 ± 0.97
DAS28-P	0.34 ± 0.14	0.33 ± 0.14	0.30 ± 0.12	0.48 ± 0.11
ESR, median (IQR)	14.21 [8.26, 23.72]	10.98 [6.64, 16.90]	23.61 [14.34, 36.71]	15.39 [8.12, 24.99]
CRP, median (IQR)	10.12 [4.45, 26.50]	6.62 [3.61, 12.91]	31.02 [14.46, 58.97]	10.19 [4.02, 24.50]
SJC28, median (IQR)	1.50 [0.60, 3.00]	1.04 [0.43, 2.25]	1.97 [0.83, 3.71]	2.48 [1.34, 4.50]
TJC28, median (IQR)	2.00 [1.00, 4.23]	1.56 [0.76, 2.62]	2.11 [1.11, 3.82]	7.65 [5.78, 10.28]
Tender-swollen difference	0.56 [-0.36, 1.89]	0.38 [-0.38, 1.07]	0.24 [-1.00, 1.33]	4.29 [2.68, 6.63]
Large Joint Index SJC	0.00 [0.00, 0.25]	0.00 [0.00, 0.14]	0.03 [0.00, 0.33]	0.20 [0.00, 0.60]
Large Joint Index TJC	0.50 [0.08, 1.04]	0.29 [0.00, 0.72]	0.50 [0.09, 1.00]	1.81 [1.19, 2.36]
VAS - GH patient	35.55 ± 16.74	30.11 ± 13.73	36.98 ± 16.22	51.46 ± 16.30
CBC parameters				
Neutrophil-to-lymphocyte Ratio	0.58 ± 0.15	0.50 ± 0.09	0.74 ± 0.13	0.55 ± 0.11
Platelet-to-lymphocyte ratio	2.18 ± 0.16	2.12 ± 0.13	2.33 ± 0.14	2.13 ± 0.14
Platelet-neutrophil count	1. 36 ± 0.19	1.27 ± 0.14	1.55 ± 0.16	1.32 ± 0.15
SII	2.84 ± 0.24	2.72 ± 0.18	3.08 ± 0.18	2.79 ± 0.20
Haemoglobin	8.35 ± 0.79	8.55 ± 0.70	7.94 ± 0.82	8.35 ± 0.77
Lymphocyte-to-monocyte ratio	0.61 ± 0.12	0.65 ± 0.10	0.51 ± 0.10	0.65 ± 0.10
ALAT	27.58 ± 18.01	26.67 ± 11.11	26.29 ± 14.90	32.85 ± 33.96
Medication				
Medication data available, n	492	295	111	86
csDMARD, n (%)	480 (97.6)	286 (96.9)	111 (100)	83 (96.5)
b/tsDMARD, n (%)	288 (58.5)	161 (54.6)	64 (57.7)	63 (73.3)
TNF inhibitor, n (%)	244 (49.6)	143 (48.5)	52 (46.8)	49 (57.0)
IL-6 receptor inhibitor, n (%)	81 (16.5)	32 (10.8)	17 (15.3)	32 (37.2)
JAK inhibitor, n (%)	13 (2.6)	4 (1.4)	2 (1.8)	7 (8.1)
T-cell costimulation modulator, n (%)	38 (7.7)	7 (2.4)	12 (10.8)	19 (22.1)
B-cell depletion / anti-CD20, n (%)	46 (9.3)	15 (5.1)	15 (13.5)	16 (18.6)
IL-1 inhibitor, n (%)	3 (0.6)	0 (0.0)	1 (0.9)	2 (2.3)
Glucocorticoids, n (%)	307 (62.4)	145 (49.2)	94 (84.7)	68 (79.1)

ALAT, Alanine aminotrasferase; b/tsDMARD, biological or targeted synthetic disease-modifying antirheumatic drug; CBC, complete blood count; CRP, C-reactive protein; csDMARD, conventional disease-modifying antirheumatic drug; DA, disease activity; DAS28, Disease Activity Score in 28 Joints; ESR, erythrocyte sedimentation rate; IL, interleukin; SII, systemic immune-inflammation index; SJC, swollen joint count; TJC, tender joint count; TNF, tumour necrosis factor; VAS-GH, Visual Analogue Scale for Global Health; JAK Inhibitor, Januse Kinase Inhibitors.

Radiographic progression parameters are also presented. All variables are presented as mean (SD) unless mentioned otherwise. Medication mechanism-of-action percentages were calculated among patients with available medication data in the radiographic analysis cohort. Categories are not mutually exclusive, as patients may have received multiple b/tsDMARD classes over follow-up.

### Definition of disease activity expression profiles

Three clusters were identified as the optimal solution statistically as well as based on clinical relevance as compared to 2 and 4 clusters (Supplementary Fig S1). Based on silhouette scores, stability, and clinical interpretability, the 3-cluster solution was selected as the primary solution. Resulting distinct disease activity expression profiles are depicted in Figure 1, and general patient and disease characteristics are described in Table 1. The largest group showed low-to-moderate disease activity (DAS28 = 2.62 [0.73]), with relatively similar levels across clinical, lab, and patient-reported measurements relatively close to the overall mean (n = 646; 55.3%). This cluster was designated as ‘Low-Typical Disease Activity’. A second cluster (n = 330; 28.3%: DAS28, 3.41 [0.84]) demonstrated markedly elevated inflammatory parameters, with high SJC, ESR, CRP, and haematological inflammatory indices, including SII, NLR, and PLR, and was labelled ‘Systemic inflammatory disease activity’. The third cluster (n = 191; 16.4%, DAS28 4.04 [0.97]) was characterised by specifically high patient-reported outcomes, DAS28-P, and a notably higher tender-than-swollen joint count, suggesting a greater subjective disease burden. This group was designated as ‘Symptom-dominant disease activity’. In the subset with available medication data, treatment exposure differed across profiles, with higher treatment use observed in the Symptom-dominant profile.

**Figure 1:**
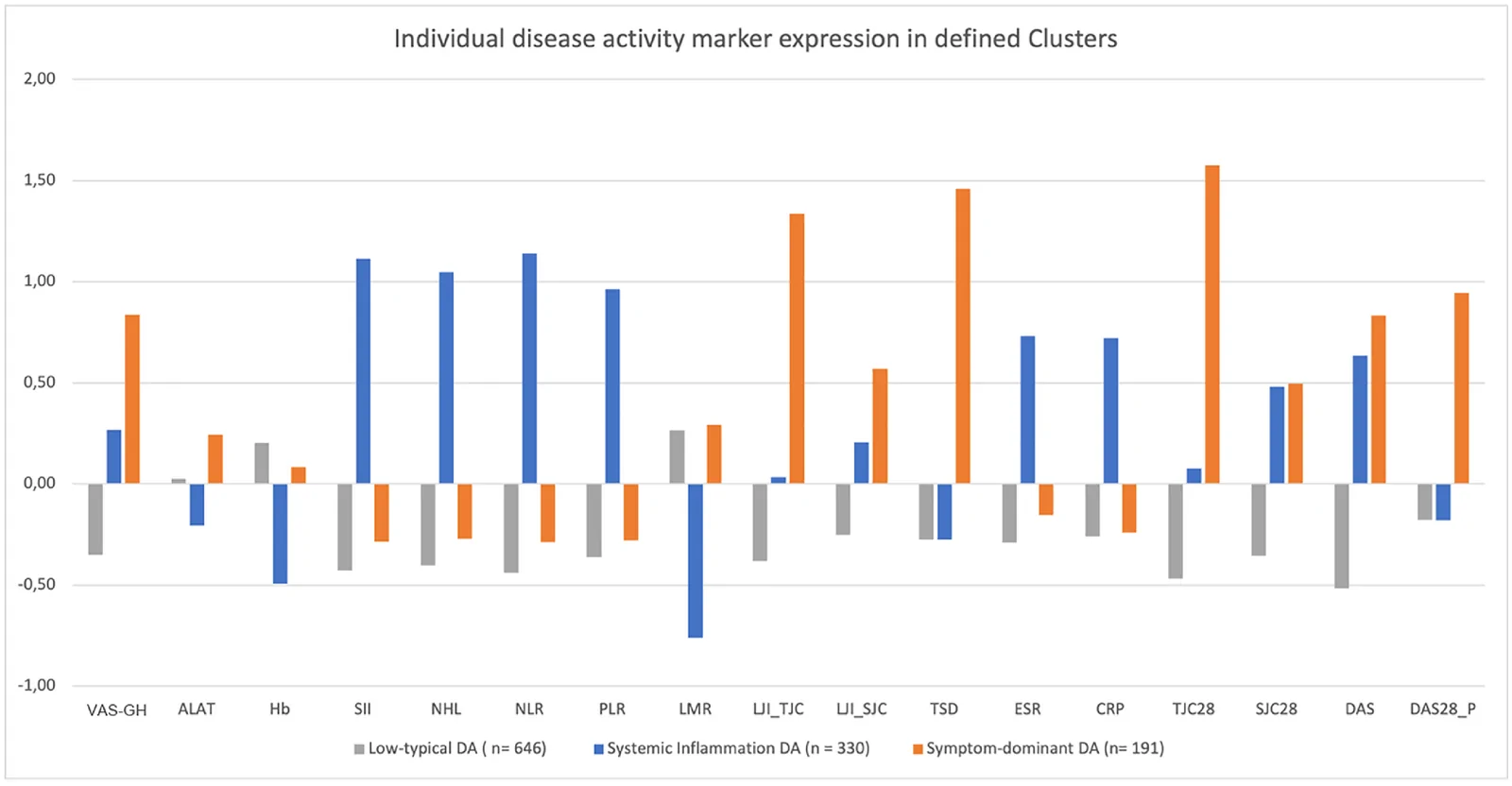
This figure represents the distribution of the 17 disease activity markers investigated relative to the overall mean for the 3 defined disease activity expression clusters: Low-Typical Disease Activity, systemic inflammatory disease activity, and Symptom-dominant disease activity. CRP, C-reactive protein; DAS, Disease Activity Score; DAS28, Disease Activity Score in 28 Joints; ESR, erythrocyte sedimentation;VAS-GH, Visual Analog Scale for General Health; Hb, haemoglobin; ALAT, alanine aminotransferase; LJI_TJC, LJI_SJC, large tender and swollen joint; complete blood count components (LMR: lymphocyte-to-monocyte ratio; NHL, neutrophil to haemoglobin ratio; NLR, neutrophil-to-lymphocyte ratio; PLR, platelet-to-lymphocyte ratio; SII: platelet counts × neutrophil counts/lymphocyte counts) tender and swollen 28 joint counts (TJC28 and SJC28); SII, systemic immune-inflammation index; TSD, tender and swollen joint count difference; DAS28 score (DAS) and DAS28 pain score^3^ (DAS28-P = [0.56 × TJC + 0.014 × VAS − GH]/DAS28ESR).

In sensitivity analyses at visit level and using relative measures of disease activity markers, cluster solutions were consistent with the primary analysis (Supplementary Fig S3). The predefined stability criterion was met: 76.3% of patients had an expression profile consistency of ≥75% across visits, and the average observed consistency rate was 88.2% (Q1-Q3: 75%-100%). This exceeded our predefined stability criterion, supporting the interpretation of disease activity expression profiles as a patient-level trait rather than purely visit-level states (Supplementary Fig S2).

The most important variables in cluster classification, based on decreases in mean accuracy and Gini, were TJC28 and NLR (Fig 2). These were followed by LJI_TJC, TSD, and SII, suggesting that both subjective joint assessments and inflammatory markers were key in differentiating patient subgroups. Traditional inflammation markers such as ESR and CRP also contributed, though to a lesser extent. In contrast, variables such as VAS_PAT, DAS28_P, and ALAT showed low importance for cluster assignment. These results were also consistent in sensitivity analyses.

**Figure 2:**
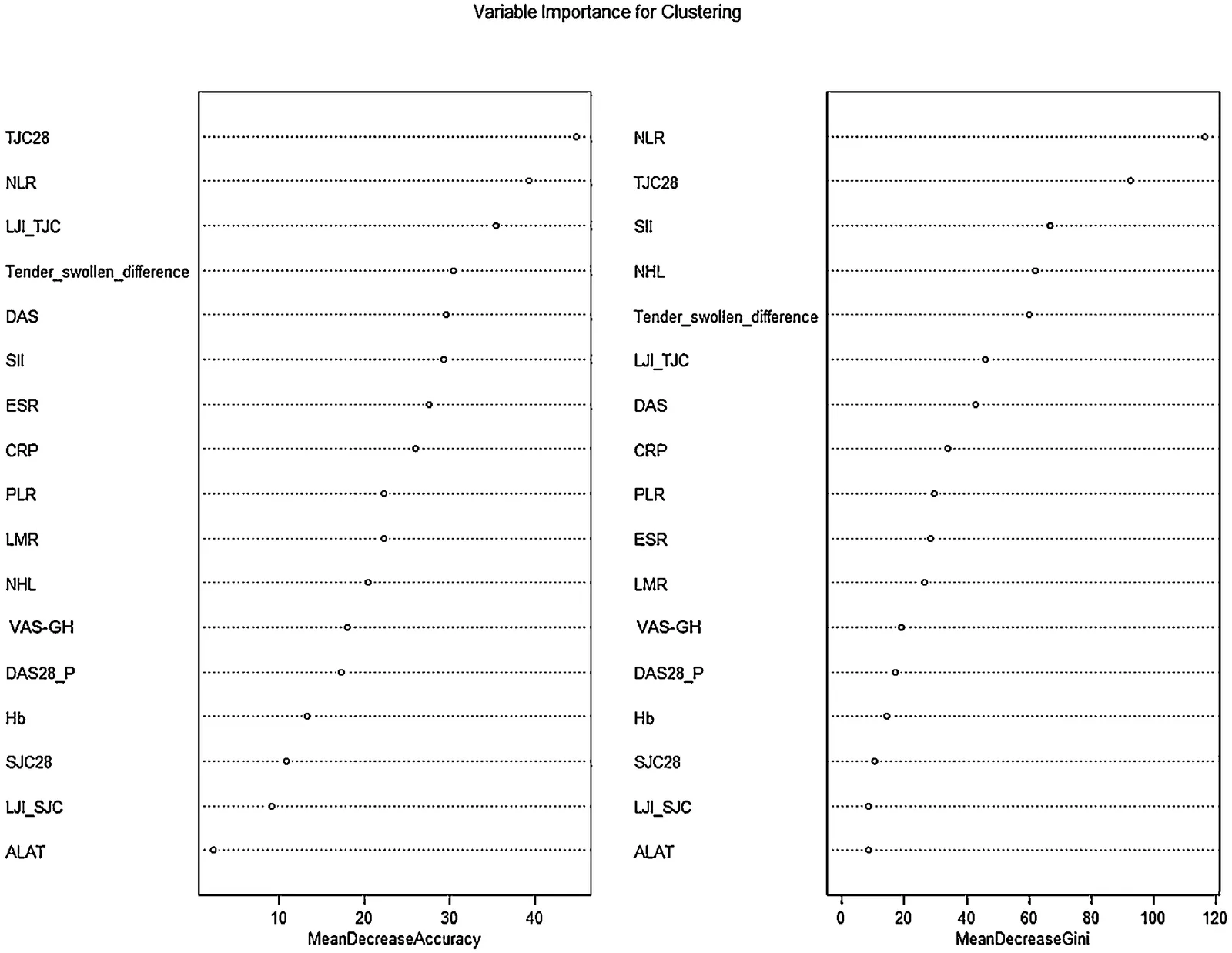
Variable importance thorough mean decrease in accuracy and Gini impurity in a random forest model for predicting cluster membership. CRP, c-reactive protein; DAS, Disease Activity Score; DAS28, Disease Activity Score in 28 Joints; ESR, erythrocyte sedimentation; Hb, haemoglobin; ALAT, alanine aminotransferase; LJI_TJC, LJI_SJC, large tender and swollen joint count; VAS-GH Visual Analog Scale for General Health; complete blood count components (NHL, neutrophil to haemoglobin ratio; NLR, neutrophil-to-lymphocyte ratio; PLR, platelet-to-lymphocyte ratio; LMR, lymphocyte-to-monocyte ratio; SII, platelet counts × neutrophil counts/lymphocyte counts); TJC28, SJC28, tender and swollen 28 joint counts; TSD, tender and swollen joint count difference; DAS28 score (DAS) and DAS28 pain score^3^ (DAS28-P = [0.56 × TJC + 0.014 × VAS − GH]/DAS28ESR).

### Association of disease activity expression profiles with radiographic progression of joint damage

As expected, the presence of baseline structural damage (odds ratios [OR] = 7.90; 95% CI, 3.30-18.90; *P* < .001), longer time between radiographic assessments (OR = 1.03; 95% CI, 1.02-1.04; *P* < .001) and higher DAS28 (OR = 1.40; 95% CI, 1.03-1.92; *P* = .032) were significantly associated with increased risk of radiographic progression.

Cluster membership was related to progression risk and, importantly, also influenced the effect of DAS28 on progression risk (*P* < .032). A slightly stronger effect of the DAS28 on progression risk was observed for the Systemic inflammatory cluster (OR = 1.14; 95% CI, 0.67-1.93; *P* = .63) compared with the Low-typical cluster. However, in the Symptom-dominant cluster, the DAS28 interaction with progression risk was weaker (OR = 0.54; 95% CI, 0.32-0.89; *P* = .015) than in the Low-typical cluster (Table 2).

**Table 2:** Mixed-effects model of disease progression and effect of disease activity expression membership

	Full model		
Predictor variable	Odds Ratio	95% CI	*P* value
Step 1: Baseline effects			
Intercept	0.031	0.009-0.105	<.001*
Baseline structural damage	7.90	3.30-18.90	<.001*
Time between radiographic assessments	1.03	1.02-1.04	<.001*
DAS28	1.40	1.03-1.92	.032
Step 2: Cluster effects			
Systemic inflammatory DA cluster	5.22	0.74-36.7	.096
Symptom-dominant DA cluster	5.88	0.86-46.3	.079
Step 3: DAS28 interaction			
DAS28 × Systemic inflammatory DA	1.14	0.67-1.93	.63
DAS28 × Symptom-dominant DA	0.54	0.32-0.89	.015*

DA, disease activity; DAS, Disease Activity Score; DAS28, Disease Activity Score in 28 Joints.

⁎ Statistical significance is indicated at the p < 0.05 level.

Figure 3 graphically displays the effect modification of disease activity, categorised by the DAS28 categories (remission, low-, moderate-, and high) by plotting predicted and observed progression probability stratified by cluster membership [30]. Within the Low-typical cluster (left panel), the predicted risk of progression increases with higher DAS categories as expected, although the absolute risks remain modest. In contrast, patients in the Systemic inflammatory cluster (centre panel) consistently demonstrate a higher progression risk, with a similar increase with higher DAS28 categories compared with the Low-Typical Disease Activity group. In the Symptom-dominant cluster (right panel), the predicted absolute risks appeared lower across DAS28 categories. Given uncertainty around the cluster main effect, the absolute risk pattern should be interpreted cautiously.

**Figure 3:**
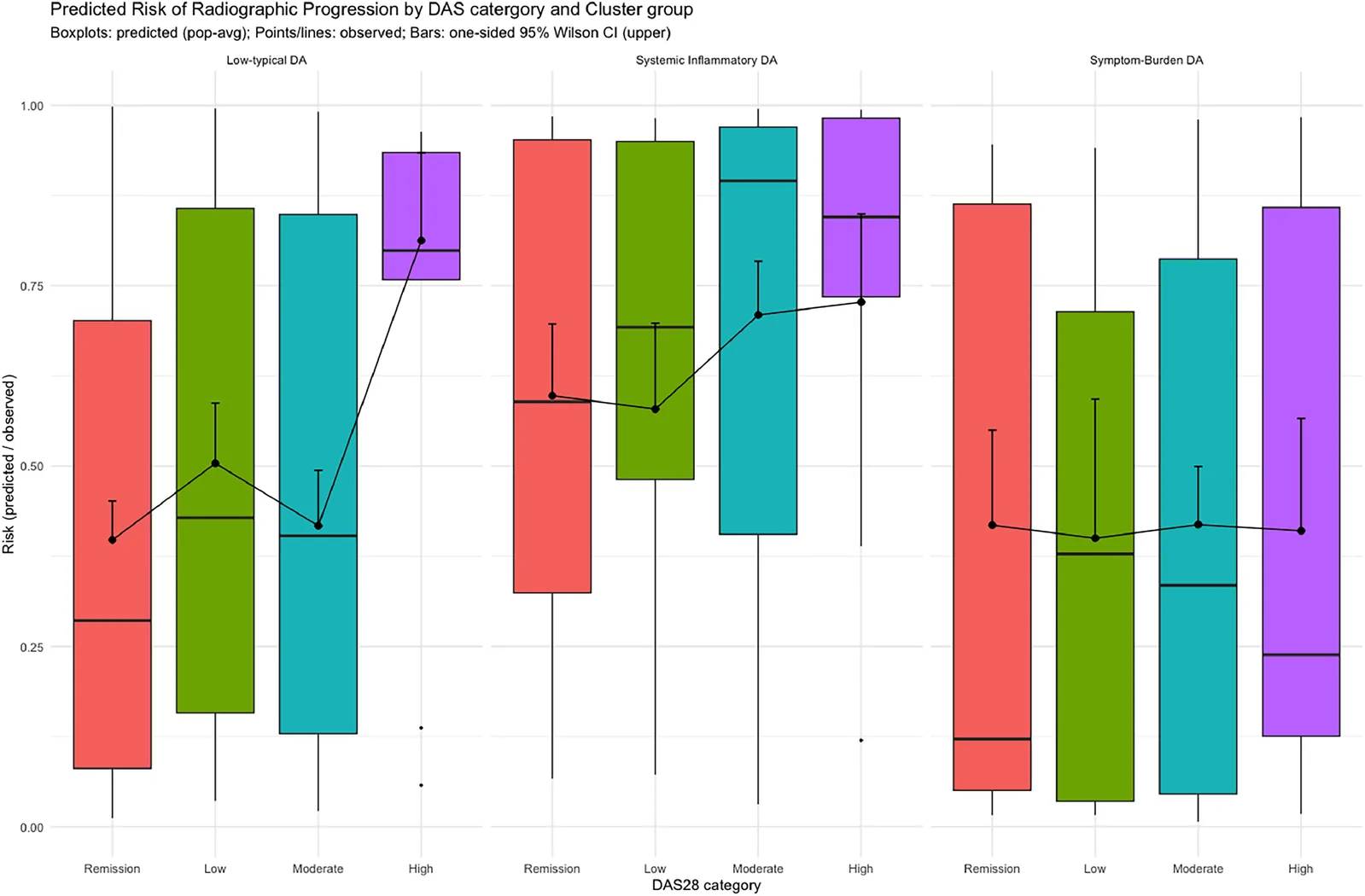
The figure plots the estimated joint damage progression risk with increasing level of disease activity, separately for the identified disease activity expression clusters. The 3 observed clusters are shown in interaction with the 4 DAS28 categories (remission, low, moderate, and high). Superimposed red dots represent the observed proportion of patients who experienced radiographic progression in each category, and red lines connect these proportions across DAS categories within each cluster. Points indicate observed proportions; vertical error bars represent 1-sided 95% Wilson binomial confidence intervals. DA, disease activity; DAS, Disease Activity Score; DAS28, Disease Activity Score in 28 Joints.

Although the 3-cluster solution was retained as the primary clustering framework, we explored, as an ad hoc analysis, whether objective joint swelling in the Symptom-dominant cluster further stratified progression risk. As it unexpectedly presented relatively high DAS28, SJC28, and ESR values, this cluster was further subdivided to distinguish individuals with an SJC28 of 2 or more and those with lower SJC28. This analysis demonstrated that patients with ≥2 swollen joints had an increased risk of radiographic progression compared with those with <2 swollen joints, although this risk remained lower than that observed in the Systemic inflammatory cluster. Moreover, higher DAS28 was not associated with radiographic progression in both subgroups (Supplementary Fig S4).

### Prediction of cluster membership

Table 3 reports the ORs and 95% CIs for variables at the first visit from the multinomial logistic regression, with the Low-typical cluster as the reference group. The probability of belonging to the Systemic inflammatory cluster mainly increased with laboratory-based markers of systemic inflammation: higher NLR (OR = 2.96; 95% CI, 1.59-5.49), lower LMR (OR = 0.59; 95% CI, 0.44-0.80), and lower Hb (OR = 0.72; 95% CI, 0.55-0.95) values. Higher DAS28-P values were associated with reduced risk of belonging to this cluster (OR = 0.62; 95% CI, 0.42-0.92). In addition, older age presented increased odds of belonging to this cluster (OR = 1.35; 95% CI, 1.07-1.71).

**Table 3:** Multivariable logistic regression models showing the association between standardised predictors and cluster membership, presented as odd ratios (ORs) with 95% CIs

Predictor	Systemic inflammatory DA cluster OR (95% CI)	*P* value	Symptom-dominant DA cluster OR (95% CI)	*P* value
Intercept	0.33 (0.23-0.46)	<.001*	0.12 (0.08-0.20)	<.001*
Age	**1.35 (1.07-1.71)**	.012*	1.08 (0.83-1.41)	.553
Gender	1.38 (0.82-2.32)	.221	0.94 (0.51-1.73)	.846
LJI-TJC	1.11 (0.80-1.54)	.520	1.49 (1.11-1.99)	.007*
CRP	0.79 (0.59-1.06)	.118	0.82 (0.59-1.15)	.260
DAS	1.74 (0.93-3.26)	.084	4.46 (1.79-11.09)	.001*
DAS28-P	0.62 (0.42-0.92)	.016*	3.14 (1.56-6.32)	.001*
ESR	1.05 (0.67-1.65)	.817	0.88 (0.53-1.45)	.611
Haemoglobin	0.72 (0.55-0.95)	**.**019*	0.95 (0.70-1.28)	.729
LJI SJC	1.03 (0.79-1.34)	.816	1.17 (0.91-1.52)	.228
LMR	0.59 (0.44-0.80)	<.001*	1.30 (0.93-1.81)	.125
NLR	2.96 (1.59-5.49)	<.001*	1.07 (0.57-2.01)	.841
PLR	1.15 (0.79-1.66)	.471	0.67 (0.44-1.03)	.070
SII	1.43 (0.75-2.72)	.276	1.93 (0.93-3.99)	.077
SJC28	0.82 (0.61-1.11)	.197	0.70 (0.49-0.99)	.046*
TJC28	1.00 (0.73-1.36)	.998	0.96 (0.65-1.43)	.855
TSD	1.16 (0.90-1.49)	.241	1.26 (0.94-1.69)	.124
Patient VAS	1.21 (0.90-1.64)	.204	0.90 (0.64-1.25)	.529

CRP, C-reactive protein; DAS, Disease Activity Score; DAS28, Disease Activity Score in 28 Joints.ESR, erythrocyte sedimentation; Hb, haemoglobin; ALAT, alanine aminotransferase; LJI_TJC, LJI_SJC, large tender and swollen joint count; complete blood count components (LMR, lymphocyte-to-monocyte ratio; NHL, neutrophil to haemoglobin ratio; NLR, neutrophil-to-lymphocyte ratio; PLR, platelet-to-lymphocyte ratio; SII: platelet counts × neutrophil counts/lymphocyte counts); TJC28, SJC28, tender and swollen 28 joint counts; TSD, tender and swollen joint count difference; VAS, Visual Analogue Scale. DAS28 score (DAS) and DAS28 pain score]^3^ (DAS28-P = [0.56*TJC + 0.014*VAS − GH]/DAS28ESR).

⁎ Statistical significance is indicated at the p < 0.05 level.

In contrast, the probability of belonging to the Symptom-dominant cluster increased with higher baseline DAS (OR = 4.46; 95% CI, 1.79-11.09), higher DAS28-P (R = 3.14; 95% CI, 1.56-6.32), and higher LJI-TJC (OR = 1.49; 95% CI, 1.11-1.99). Higher SJC28 was associated with a lower probability of belonging to this cluster (OR = 0.70; 95% CI, 0.49-0.99; *P* = .046). Based on individual predictor measurements obtained at the first visit, 72.7% of patients were correctly classified into their respective disease activity cluster. This exceeded the majority-class no-information rate of 52.5%, based on assigning all patients to the largest profile, and the random proportional baseline of 39.3%. Positive predictive values were 74.7% for Low-typical, 71.3% for Systemic inflammatory, and 67.5% for Symptom-dominant profiles, exceeding their corresponding a priori probabilities of 52.5%, 28.6%, and 18.8%, respectively (Supplementary Table S3).

## DISCUSSION

This study investigated the heterogeneity in disease activity expression among patients with RA and evaluated its possible clinical relevance by investigating their relationship with disease progression independent of disease activity measured with a typical disease activity index.

Based on the available laboratory and clinical disease activity variables in our cohort, we identified 3 distinct expression profiles: (i) the Systemic inflammatory cluster characterised by elevated systemic inflammatory markers and haematological indices; (ii) a Symptom-dominant cluster marked by high patient-reported symptoms despite relatively low inflammation; and (iii) and a Low-typical cluster with overall lower inflammatory and clinical activity [31,32]. Using individual observations of patients, we found that, on average, 88% of observations within a patient were classified in the same cluster, suggesting that these expression profiles represent patients’ traits rather than characteristics that vary over time. The high within-patient cluster stability further supports the interpretation of disease activity expression profiles as patient-level traits with potential long-term prognostic value.

The Systemic inflammatory cluster showed an increased risk of radiographic progression independent of DAS28, suggesting that systemic inflammation may drive joint damage beyond conventional disease activity measures, whereas the Symptom-dominant cluster likely reflects pain-dominant mixed disease mechanisms [32]. Importantly, in this last cluster, no relation between a higher DAS28 and joint damage progression was found, suggesting that the DAS28 does not adequately reflect the RA disease activity process in this subgroup. Thus, the finding does not imply that these patients are uniformly at low structural risk, but rather that DAS28 is less informative about structural progression risk. This further confirms the relevance of the identified profiles and supports the notion that RA disease activity is not expressed uniformly across patients.

The haematological parameters included in this study were selected because of their previously demonstrated associations with disease activity and treatment response in RA. Ratios such as LMR, NLR, and PLR have consistently been shown to distinguish active disease from remission, a pattern also reflected in our cluster structure [33]. As objective laboratory biomarkers of systemic inflammation, these measures capture inflammatory burden driven by synovial immune activation and are less susceptible to subjectivity than composite or symptom-based disease activity measures. Moreover, as ratio-based indices, they tend to be more stable than absolute leukocyte counts and may be less influenced by short-term biological or sampling variability. Based on our results, they may indeed be important additional markers for interpreting disease activity in patients with RA. Results may also warrant a further exploration of CBC markers in the context of RA.

In addition, the SII, which integrates neutrophil, lymphocyte, and platelet counts, has emerged as a comprehensive marker reflecting the balance between inflammatory and immune responses. Studies have shown that SII and related CBC-derived indices are associated with disease activity, prognosis, and treatment outcomes across several chronic inflammatory and immune-mediated conditions, highlighting their broader clinical relevance as accessible indicators of systemic inflammation [32], [33].

An unexpected finding was the relatively high DAS28 and SJC28 in the Symptom-dominant subgroup. The exploratory SJC28 subanalysis was not intended to redefine the clustering solution, but to assess whether objective joint swelling further stratified structural progression risk within this profile. Splitting this subgroup into patients with SJC <2 and SJC ≥ 2 revealed a higher risk of radiographic progression in the SJC ≥2 group, although in neither subgroup was a clear relationship between higher DAS28 and progression observed. This suggests that DAS28, as a composite CDAI, is not a good indicator of inflammatory RA disease activity in this subgroup. A 4-cluster solution was evaluated during cluster selection, but the additional split mainly subdivided the Low-typical profile according to overall marker levels and did not provide a statistically or clinically stronger solution than the more parsimonious 3-cluster framework. Together, these findings suggest that SJC28 may provide useful within-profile risk stratification, whereas the 3-cluster solution remains the preferred framework for describing overall disease activity expression patterns.

This study has several strengths. It is based on a large, long-term RA cohort with a median follow-up of approximately 15 years, over 130,000 clinical visits, and over 7000 radiographic assessments, enabling robust evaluation of both disease activity patterns and structural progression. The use of routinely collected clinical and laboratory markers enhances the applicability of our findings to real-world practice, and the convergence of clustering, mixed-effects modelling, and multinomial regression provides internal consistency across independent analytic approaches.

However, several limitations should be acknowledged. First, this was an observational, single-centre study, and residual confounding by unmeasured factors (eg, comorbidities, treatment exposure, and psychosocial variables) cannot be excluded. Treatment decisions are influenced by disease severity and may in turn affect inflammatory markers [34]. To explore potential confounding by treatment, we described available by disease activity expression profiles and performed sensitivity analyses adjusting the mixed-effects models for active biological or targeted synthetic disease-modifying antirheumatic drug, glucocorticoid, and interleukin 6 (IL-6) inhibitor exposure over time. However, adjustment for treatment exposure did not materially change the main conclusion: the DAS28 × Symptom-dominant interaction remained statistically significant with virtually unchanged effect estimates across all treatment-adjusted models, whereas the DAS28 × Systemic inflammatory interaction remained nonsignificant. The main cluster-effect estimates varied somewhat after treatment adjustment, particularly after IL-6 inhibitor adjustment, but the overall interpretation of the interaction analyses was unchanged (Supplementary Table S4). Residual confounding by indication therefore cannot be excluded [35].

Second, radiographic progression was derived from routinely collected radiology reports using a text-mining pipeline rather than from standardised central radiographic scoring. Although the pipeline showed good cross-validated performance in the larger manually labelled dataset, agreement with expert consensus in the smaller validation subset was moderate, reflecting the inherent ambiguity of routine radiology report descriptions of progression. Some outcome misclassification therefore cannot be excluded. However, this misclassification would most likely have attenuated rather than inflated associations with disease activity expression profiles, and the observed associations with established predictors of progression support the clinical plausibility of the outcome definition. Third, NLR and related haematological indices reflect systemic inflammation rather than RA-specific pathways and may be influenced by infections, corticosteroid use, genetic predispositions to inflammation, or other comorbid conditions. Fourth, initial visit data are not always time of diagnosis, but the time of first visit, after a refall from the second line. This could impact the prediction of our cluster subgroups, and it would be interesting to see these profiles predicted with multiple visits. Fourth, external validation in independent RA cohorts and in different healthcare settings is required to confirm the generalisability of these clusters and prediction models.

Identified disease activity expression profiles may be clinically useful. For example, for the Symptom-dominant group, less frequent radiographic imaging may be required, with more focus on psychological intervention treatments (eg cognitive behavioural therapy and pain coping skills training) and using other modes of RA joint-related disease activity, like ultrasound [[15], [30]]. Patients with a systemic inflammatory profile may benefit from close monitoring of disease activity, using, for example, the DAS28 with a relatively strict target (ie, remission); also, more systemic evaluation may be of benefit. For both these subgroups, assessment of and control of comorbidities, such as fibromyalgia and cardiovascular disease, may be indicated. This should be investigated further in future research. Also, to use identified profiles in care, they should be predictable prospectively. Indeed, we found that cluster membership could be predicted by the available markers at a patient’s first visit. This prediction may be further improved using more than 1 measurement moment or information on comorbidity and treatment.

Finally, while we treated disease activity expression profiles as stable traits, some patients may switch clusters over time, particularly in response to treatment intensification or remission induction strategies, which warrants future longitudinal trajectory analyses.

In conclusion, this study identifies clinically meaningful RA disease activity profiles, including a Systemic inflammatory phenotype, a Symptom-dominant phenotype, and a Low-typical phenotype with lower progression risk. Haematological ratios (NLR and LMR), together with DAS28 components and joint counts, characterise these profiles and help explain how disease activity translates into structural damage. Overall, these findings support a phenotype-based approach to risk stratification, monitoring, and treatment, integrating both inflammatory burden and symptom expression when guiding therapy and monitoring progression.

## CRediT authorship contribution statement

**Chiara Ripepi:** Writing – original draft, Methodology, Investigation, Formal analysis. **Bram van Es:** Writing – review & editing, Data curation. **Janneke Tekstra:** Writing – review & editing, Investigation, Data curation. **Julia Spierings:** Writing – review & editing, Supervision, Conceptualization. **Jacob M. van Laar:** Writing – review & editing, Supervision, Project administration. **Saskia Haitjema:** Writing – review & editing, Supervision, Data curation, Conceptualization. **Paco M.J. Welsing:** Writing – review & editing, Validation, Supervision, Methodology, Formal analysis, Conceptualization.

## Competing interests

All authors declare they have no competing interests.

## Appendix Supplementary materials

Image, application 1

## Footnotes

Supplementary material associated with this article can be found in the online version at doi:10.1016/j.ero.2026.100243.
