# Supplementary material for: Individual patient disease activity expression profiles modify the relation between disease activity and structural damage progression in RA
Source: EULAR Rheumatol Open. 2026 Sep 30;2(4):100243. doi: 10.1016/j.ero.2026.100243 (PMC13657163; doi:10.1016/j.ero.2026.100243)
Supplement: Supplementary file 1 [file mmc1.docx]

Supplementary material

Supplementary Table S1: Demographic and clinical characteristics of RA, categorized by their cluster affiliation. Radiographic progression paraments are also presented.

|  |  | | Symptom Dominant DA Cluster (191) | |
| --- | --- | --- | --- | --- |
|  | Symptom Dominant DA (191) | *Subgroup Median SJC28 <2 (88)* | | *Subgroup Median SJC28 >2 (103)* |
| Age | 53.67 ± 13.37 | 52.97 ± 13.71 | | 54.28 ± 13.11 |
| Female sex, n (%) | 150 (78.5%) | 67 (76.1%) | | 83 (80.6%) |
| Baseline damage (%) | 87 (47.8%) | 35 (44.3%) | | 37 (45.7%) |
| RF positive n (%) | 137 (88.4%) | 62 (70.5%) | | 75 (72.8%) |
| Follow-up years | 13.72 ± 6.43 | 15.26 [11.05, 19.08] | | 14.30 [7.85, 18.57] |
| Follow-up visits, median (IQR) | 110 [47, 216] | 120.50 [51.00, 205.75] | | 98.00 [41.50, 216.00] |
| Number of X-rays | 4.00 [1.00, 8.00] | 4.00 [1.00, 8.00] | | 3.00 [1.00, 8.00] |
| DAS28-ESR | 4.04 ± 0.97 | 3.61 ± 0.78 | | 4.36 ± 0.98 |
| DAS28-P | 0.48 ± 0.11 | 0.50 ± 0.11 | | 0.46 ± 0.11 |
| ESR, median (IQR) | 15.39 [8.12, 24.99] | 14.11 [8.28, 21.98] | | 16.88 [8.13, 28.11] |
| CRP, median (IQR) | 10.19 [4.02, 24.50] | 9.40 [3.91, 21.33] | | 10.67 [3.99, 28.05] |
| SJC28, median (IQR) | 2.48 [1.34, 4.50] | 1.28 [0.54, 2.00] | | 4.27 [2.86, 6.50] |
| TJC28, median (IQR) | 7.65 [5.78, 10.28] | 7.17 [5.42, 9.01] | | 7.88 [6.00, 10.73] |
| Tender-swollen Difference | 4.29 [2.68, 6.63] | 5.39 [3.95, 8.51] | | 3.12 [1.61, 5.06] |
| Large Joint Index SJC | 0.20 [0.00, 0.60] | 0.14 [0.00, 0.26] | | 0.42 [0.06, 0.86] |
| Large Joint Index TJC | 1.81 [1.19, 2.36] | 1.82 [1.25, 2.14] | | 1.67 [1.04, 2.50] |
| VAS Patient | 51.46 ± 16.30 | 50.68 ± 16.10 | | 51.83 ± 16.72 |
| *CBC parameters* |  |  | |  |
| Neutrophil-to-lymphocyte Ratio | 0.55 ± 0.11 | 0.54 ± 0.12 | | 0.55 ± 0.11 |
| Platelet-to-lymphocyte Ratio | 2.13 ± 0.14 | 2.11 ± 0.14 | | 2.15 ± 0.14 |
| Platelet-neutrophil count | 1.32 ± 0.15 | 1.29 ± 0.24 | | 1.32 ± 0.29 |
| SII | 2.79 ± 0.20 | 2.78 ± 0.19 | | 2.80 ± 0.20 |
| Haemoglobin | 8.35 ± 0.77 | 8.40 ± 0.78 | | 8.31 ± 0.75 |
| Lymphocyte-to-monocyte Ratio | 0.65 ± 0.10 | 0.65 ± 0.10 | | 0.65 ± 0.10 |
| ALT | 32.85 ± 33.96 | 32.94 ± 45.60 | | 33.13 ± 20.91 |

All Variables are presented as mean (sd) unless mentioned otherwise)

Supplementary table S2: Mixed effects model of disease progression and effect of disease activity-expression membership.

|  | Model 1 |  |  | Model 2 |  |  |
| --- | --- | --- | --- | --- | --- | --- |
| Predictor Variable | OR | 95% CI | p-value | OR | 95% CI | p-value |
| *Step 1: Baseline Effects* |  |  |  |  |  |  |
| Intercept | 0.031 | 0.009 - 0.105 | **<0.001** | 0.030 | 0.009 - 0.107 | **<0.001** |
| Baseline structural damage | 7.90 | 3.30 - 18.90 | **<0.001** | 8.14 | 3.20 - 20.80 | **<0.001** |
| Time between radiographic assessments | 1.03 | 1.02 - 1.04 | **<0.001** | 1.03 | 1.0 - 1.04 | **<0.001** |
| DAS28 | 1.40 | 1.03 - 1.92 | 0.032 | 1.40 | 1.03 - 1.92 | **0.033** |
| *Step 2: Cluster Effects* |  |  |  |  |  |  |
| Systemic Inflammatory DA cluster | 5.22 | 0.74 - 36.7 | 0.096 | 5.40 | 0.74 - 39.3 | 0.096. |
| Symptom-dominant cluster | 5.88 | 0.86 - 46.3 | 0.079 | — | — | — |
| Symptom-dominant cluster (≥2 SJC) | — | — | — | 59.3 | 3.55 - 991 | **0.005** |
| Symptom-dominant cluster (<2 SJC) | — | — | — | 0.37 | 0.02 - 6.66 | 0.510 |
| *Step 3: DAS28 Interaction* |  |  |  |  |  |  |
| DAS28 × Inflammatory cluster | 1.14 | 0.67 - 1.93 | 0.63 | 1.14 | 0.66 - 1.92 | 0.63 |
| DAS28 x Symptom-dominant | 0.54 | 0.32 - 0.89 | **0.015** | — | — | — |
| DAS28 × Symptom-dominant (<2 SJC) | — | — | — | 0.94 | 0.44 - 2.74 | 0.87 |
| DAS28 × Symptom-dominant (≥2 SJC) | — | — | — | 0.37 | 0.20 - 0.70 | **0.002** |

Supplementary Table S3: Baseline (first visit with complete information on disease activity markers) prediction of expression profiles (n = 796 patients)

| **Observed** | **Predicted** | | |
| --- | --- | --- | --- |
|  | Low-Typical DA | Systemic Inflammatory DA | Symptom-Dominant DA |
| Low-Typical DA | **354 (74.7%)** | 39 (19.3%) | 25 (20.8%) |
| Systemic Inflammatory DA | 70 (14.8%) | **144 (71.3%)** | 14 (11.7%) |
| Symptom-Dominant DA | 50 (10.5%) | 19 (9.4%) | **81 (67.5%)** |

Supplementary Table S4: Treatment-adjusted sensitivity analyses for the association between DAS28, disease activity expression profile, and radiographic progression

| Term | Original model | Adjusted for active b/tsDMARD exposure | Adjusted for active glucocorticoid exposure | Adjusted for active IL-6 inhibitor exposure | Adjusted for active b/tsDMARD + glucocorticoid exposure |
| --- | --- | --- | --- | --- | --- |
| Symptom Dominant DA main term | 5.90 (0.82-42.69), p=0.079 | 6.06 (0.83-44.25), p=0.076 | 5.95 (0.82-43.11), p=0.078 | 6.48 (0.88-47.48), p=0.066 | 6.09 (0.83-44.43), p=0.075 |
| Systemic Inflammatory DA main term | 5.30 (0.76-37.12), p=0.093 | 5.01 (0.71-35.29), p=0.106 | 5.40 (0.77-37.88), p=0.090 | 4.44 (0.63-31.27), p=0.134 | 5.11 (0.72-36.09), p=0.102 |
| DAS28 × Symptom Dominant DA | 0.54 (0.33-0.89), p=0.015 | 0.54 (0.32-0.89), p=0.015 | 0.54 (0.33-0.90), p=0.017 | 0.54 (0.33-0.89), p=0.015 | 0.54 (0.33-0.90), p=0.017 |
| DAS28 × Systemic Inflammatory DA | 1.13 (0.67-1.91), p=0.642 | 1.15 (0.68-1.94), p=0.603 | 1.14 (0.68-1.93), p=0.613 | 1.20 (0.71-2.03), p=0.503 | 1.16 (0.68-1.96), p=0.584 |
| Active b/tsDMARD exposure | - | 0.80 (0.48-1.33), p=0.393 | - | - | 0.82 (0.49-1.37), p=0.449 |
| Active glucocorticoid exposure | - | - | 0.82 (0.46-1.49), p=0.521 | - | 0.86 (0.47-1.56), p=0.614 |
| Active IL-6 inhibitor exposure | - | - | - | 0.45 (0.19-1.08), p=0.072 | - |

Supplementary Figure S1: Gap Statistic Values Across Candidate Cluster Solutions (k = 1–10) Identifying the Optimal Number of Clusters

Supplementary Figure S2: Distribution of the Proportion of Visits Assigned to the Most Frequent Cluster per Patient

Supplementary Figure S3: This figure represents the distribution of the 17 disease activity, in relation to the DAS28 as a sensitivity analysis. Investigated relative to the overall mean for the three defined disease activity expression clusters: low-typical disease activity, systemic inflammatory disease activity and symptom-buden disease activity.

c-reactive protein (CRP), erythrocyte sedimentation (ESR), Haemoglobin (Hb), Alanine aminotransferase (ALAT), large tender and swollen joint count (LJI_TJC, LJI_SJC), complete blood count components (SII: platelet counts × neutrophil counts/lymphocyte counts, NHL: Neutrophil to haemoglobin ratio, NLR: Neutrophil to lymphocyte ratio, PLR: Platelet to lymphocyte ratio, LMR: Lymphocyte to monocyte ratio) tender and swollen 28 joint counts (TJC28, SJC28), tender and swollen joint count difference (TSD), DAS28 score (DAS) and DAS28 pain score]^3^ (DAS28-P = (0.56*TJC + 0.014*VAS – GH)/DAS28ESR)

Supplementary Figure S4: The figure plots the **estimated joint damage progression risk** with increasing level of disease activity, separately for the identified disease activity expression clusters. The three observed clusters are shown in interaction with the 4 DAS28 categories (Remission, Low, Moderate and High). Superimposed red dots represent the observed proportion of patients who experienced radiographic progression in each category, and red lines connect these proportions across DAS categories within each cluster. Points indicate observed proportions; vertical error bars represent one-sided 95% Wilson binomial confidence intervals.
